# The EuroCropsML time series benchmark dataset for few-shot crop type classification in Europe

**DOI:** 10.1038/s41597-025-04952-7

**Published:** 2025-04-19

**Authors:** Joana Reuss, Jan Macdonald, Simon Becker, Lorenz Richter, Marco Körner

**Affiliations:** 1 Technical University of Munich, Chair of Remote Sensing Technology, Munich, 80333 Germany; 2 dida Datenschmiede GmbH, Berlin, 10827 Germany; 3 ETH Zürich, Department of Mathematics, Zürich, 8092 Switzerland; 4 Zuse Institute, Berlin, 14195 Germany

**Keywords:** Agriculture, Research data

## Abstract

We introduce EuroCropsML, an analysis-ready remote sensing dataset based on the open-source EuroCrops collection, for *machine learning (ML)* benchmarking of time series crop type classification in Europe. It is the first time-resolved remote sensing dataset designed to benchmark transnational few-shot crop type classification algorithms that supports advancements in algorithmic development and research comparability. It comprises 706683 multi-class labeled data points across 176 crop classes. Each data point features a time series of per-parcel median pixel values extracted from Sentinel-2 L1C data and precise geospatial coordinates. EuroCropsML is publicly available on Zenodo.

## Background & Summary

The availability of spatio-temporal satellite imagery and the success of data-driven modeling lead researchers to explore machine learning methods for diverse remote sensing tasks.

One key application is monitoring agricultural crop distribution worldwide, which is crucial for food security, a core *sustainable development goal (SDG)* set by the *United Nations (UN)*. With five billion hectares of agricultural land globally^1^, *crop type classification* has become a central focus in remote sensing. Consequently, numerous datasets have been compiled, such as ZueriCrop^2^ for northern Switzerland, Denethor^3^, a non-publicly available dataset for northern Germany, BreizhCrops^4^ for the French Brittany region, Pastis^5^ also covering France, Sen4AgriNet^6^ covering France and the Spanish autonomous community Catalonia, and the MGL dataset for long-term grain land changes in China^7^. Further, there is the CropHarvest^8^ collection, which is a global dataset mainly featuring binary crop-*vs*.-non-crop labels. For a comprehensive listing, we also refer to a recent overview^9^ over different available datasets focusing more broadly on earth observation data. However, as shown in Table 1, current crop classification datasets are commonly restricted to small areas within a single country. They comprise only a small number of multi-class crop labels, or include a limited number of agricultural parcels. This hinders the effective benchmarking of data-driven methods. With EuroCropsML, we aim to address the shortcomings of existing datasets and provide the first comprehensive dataset that covers many desirable aspects for benchmarking ML algorithms, with an emphasis on transfer and, in particular, few-shot learning.

**Table 1:** Comparison between EuroCropsML and other datasets with respect to availability of transnational data, number of crop type classes, covered area, number of parcels, and number of time step observations per data point.

Dataset	Transnational	# Classes	Area (in km^2^)	# Parcels	# Time steps	Period
ZueriCrop^2^	no	48	2400	116000	71	2019
BreizhCrops^4^	no	9	27200	768175	51–102	2017
CropHarvest^8^	yes	N/A^†^	global	90480	12	2020–21
Pastis^5^	no	18	4000	124000	1	2018–19
Denethor^3^	no	9	Northern Germany	4500	daily	2018–19
Sen4AgriNet^6^	yes	168	France & Catalonia	42.5 mio	5	2016–20
MGL^7^	no	7	China	11345	35	1985–20
**EuroCropsML**	yes	176	222110	706683	1–216	2021

^†^The CropHarvest class labels are not standardized between regions, 65.8 % of CropHarvest data is only binary labeled (crop vs. non-crop), it contains 348 unstructured classes in total.

*Transfer learning*, instead of directly training a model from scratch on limited target data, uses model parameters obtained from pre-training on related data as a starting point for fine-tuning on the target task. *Few-shot learning* algorithms, on the other hand, aim to enable models to perform well on tasks with very few training examples to quickly adapt to new scenarios not represented in the training data. Thus, crop type classification can benefit by leveraging detailed ground-truth data from well-documented countries to enhance classification in regions with only limited data available, potentially across varying climates and crop types. Previous studies have explored applications for few-shot learning in agriculture, including plant classification^10^, plant disease detection^11^, and crop identification using satellite imagery^12^.

*Supervised learning* algorithms for crop type classification from remote sensing data require high quality class label annotations in addition to the satellite imagery. The open-source EuroCrops dataset^13,14^ (10.5281/zenodo.8229128) provides data on agricultural parcels and crop types across the *European Union (EU)*. The data has been collected directly from farmers’ self-declarations. Its harmonized *hierarchical crop and agriculture taxonomy (HCAT)*^14,15^ makes it a valuable resource for developing and benchmarking ML algorithms across countries. The hierarchically structured and harmonized crop classes facilitate the identification of closely related crops. For instance, all cereals and flowers are distinguished from each other at a higher level: The taxonomy assigns a greater proximity to wheat and oats (both cereals) than to wheat and tulips (flower). This is advantageous for interpreting algorithmic performance and learning beyond binary crop-*vs*.-non-crop labeling. Our dataset, EuroCropsML, combines time-resolved multi-spectral Sentinel-2 L1C satellite imagery with harmonized crop type class labels from the EuroCrops reference dataset, both from the year 2021. Sentinel-2 plays a key role in EuroCropsML by providing detailed spectral and temporal data that help distinguish crops based on their growth patterns and reflectance properties. Its high spatial resolution ensures accurate identification at the field level, making it well-suited for parcel-based classification.

In summary, the strengths of our dataset are:

1. Fully annotated remote sensing crop type classification data with a coverage of diverse geographical regions representing different climate zones as well as different vegetation and agricultural practices.
2. Self-declared multi-class labels for 706683 data points classified into 176 distinct crop classes (https://github.com/dida-do/eurocropsml/blob/main/csvs/cropclasses.csv), referring to the final data after pre-processing, *cf*. Section **Level II: Data Pre-processing**. This addresses a significant limitation of some existing datasets, where labels may be less accurate and streamlined.
3. Harmonized crop labels across multiple countries that enable in-depth analyses of knowledge transfer between geographical regions based on crop taxonomy.
4. Fine-grained time series for the year 2021 with up to 216 time steps per data point. Again, this refers to the final data after pre-processing, *cf*. Section **Level II: Data Pre-processing**.
5. Provisioning of pre-built training and evaluation dataset splits ready-to-use for benchmarking crop type classification ML algorithms.

## Methods

### Country Selection

**Fig. 1:**
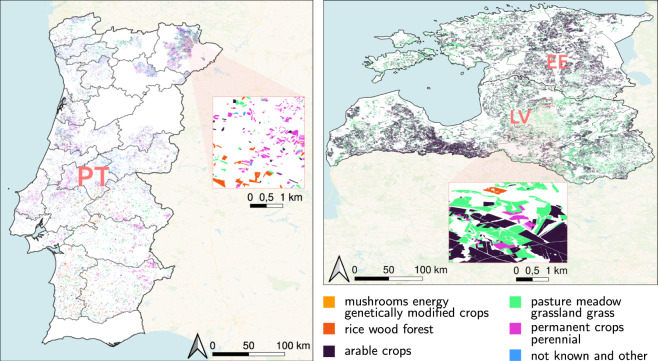
Visualization of crop fields for Estonia (EE), Latvia (LV), and Portugal (PT). The 6 shown crop-types are superclasses of the final 176 EuroCropsML classes, represent the general land cover type, and are retrieved from EuroCrops HCAT3 level 3^15^ (https://github.com/maja601/EuroCrops/blob/5c33a1133865ded9ec1a1438fc7708e84a895db5/hcat_core/HCAT3.csv). The majority of Latvia and Estonia is comprised of expansive clusters of arable crops and pasture meadow grassland grass. In contrast, Portugal presents a more diverse distribution among the various classes, with crop fields exhibiting a more dispersed pattern across the country.

**Fig. 2:**
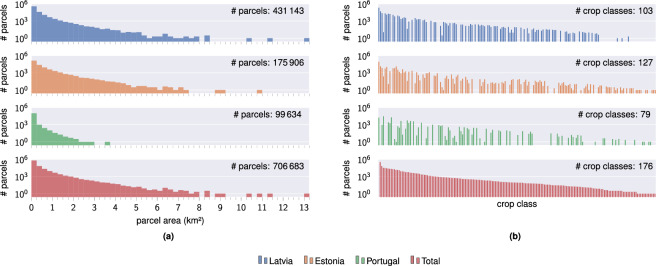
Parcel size and crop class distribution in the EuroCropsML dataset. (**a**) Number of parcels (with a log scale) of a certain size within Estonia, Latvia, Portugal, and the overall EuroCropsML dataset. The histogram bin width for the parcels sizes is 0.25 km^2^. (**b**) Number of parcels (with a log scale) of all 176 distinct EuroCropsML crop classes (HCAT3 level 6^14,15^) within Estonia, Latvia, Portugal, and the overall EuroCropsML dataset. The crop class with—by far—the largest prevalence is the meadow class.

The EuroCropsML dataset comprises three *regions of interest (ROIs)*. They are selected based on the availability of a sufficient number of labeled parcels in the reference data and to showcase locations with both similar and different climates, vegetation, and crop classes. The ROIs are Estonia, Latvia, and Portugal, as shown in Fig. 1. Latvia and Estonia are neighboring countries. In contrast, Portugal is located in a different climate zone, with different vegetation and cultivation practices, *cf*. Fig. 1. Furthermore, Fig. 2a shows the disparities in parcel size, another notable distinction between the countries’ cultivation practices. All three countries collectively provide a substantial number of overlapping and non-overlapping crop classes (*cf*. Fig. 2b). The dataset reflects the high class imbalances common in real-world crop type classification. For instance, the crop type class pasture meadow grassland grass is by far the largest class in the considered ROIs, accounting for roughly 45 % of all data points (48 % in Estonia, 50 % in Latvia, 17 % in Portugal).

### Data Collection

**Fig. 3:**
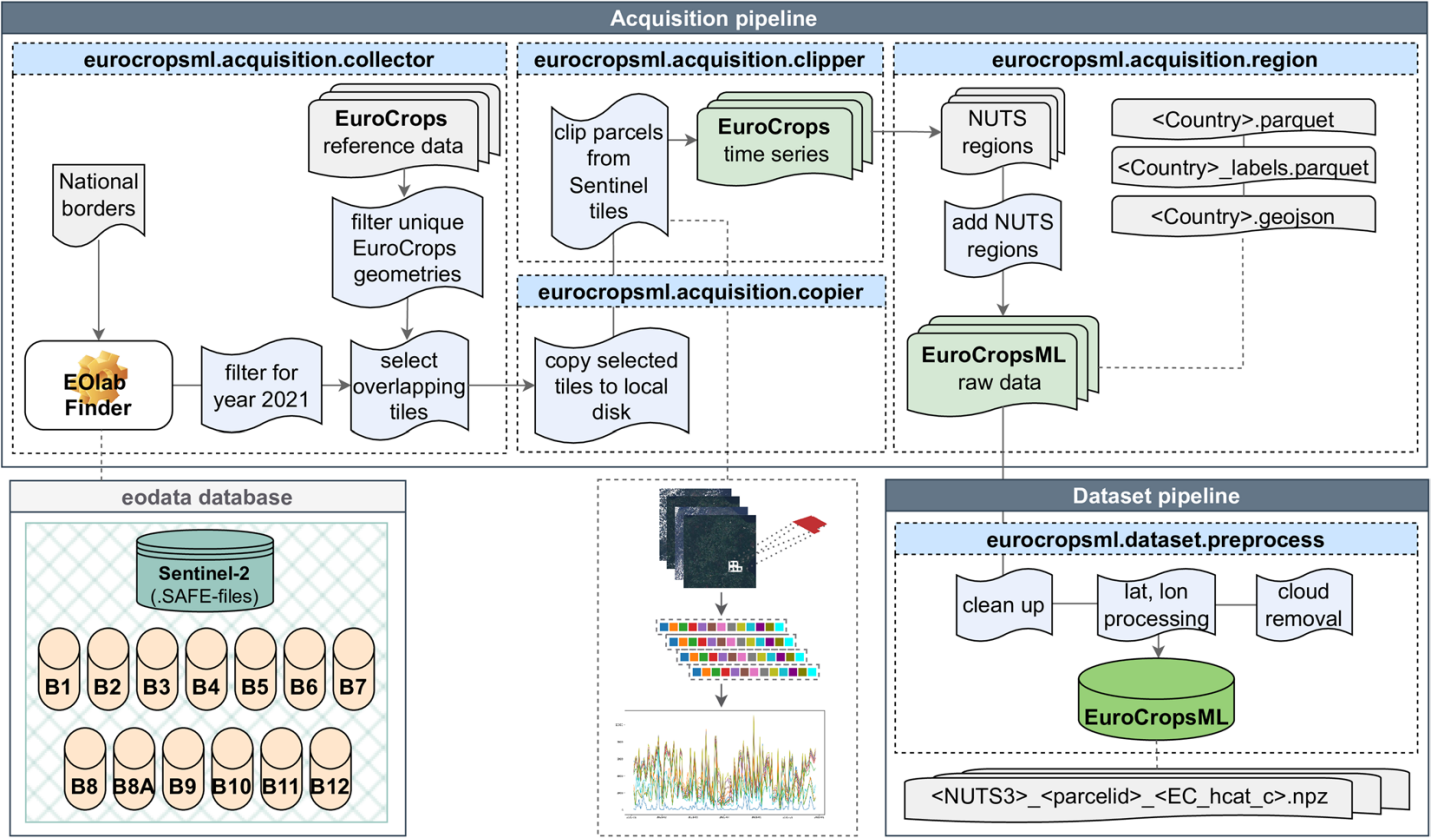
Overview of the data acquisition and pre-processing pipeline for the EuroCropsML dataset. The names in the blue headers correspond to the location and module names of the respective step in the associated Python package, available at https://github.com/dida-do/eurocropsml. A detailed view of the time series retrieval is shown in Fig. 4.

The country-specific data for EuroCropsML is generated through the process illustrated in Fig. 3 and can be broken down into two main sub-processes: data acquisition and pre-processing. Data points in the dataset correspond to one agricultural parcel and are represented by a time series of multi-spectral Sentinel-2 observation data collected throughout the year 2021. More precisely, a single observation contains the median pixel values across the parcel’s area for each of the 13 Sentinel-2 spectral bands and each of the observed points in time^15^.

#### Level I: Data Acquisition

**Collection of relevant Sentinel-2 tiles**. In the first step, the EuroCrops reference data^13^ (version 9) was aligned with Copernicus Sentinel-2 raster data tiles from the year 2021. For this, we relied on the EOLab Finder, accessible via https://finder.eo-lab.org, to identify the file paths of the Sentinel-2 .SAFE files on the EOLab platform (https://eo-lab.org/de). Please note that the EOLab Finder is no longer maintained since May 11, 2024. As a result, when processing future data, please refer to its successor, the EOLab Data Explorer, accessible via https://explore.eo-lab.org, instead. We first collected all tiles that overlap with the land surface of the given country for 2021, before mapping the collection to each agricultural parcel individually. It should be noted that some EuroCrops reference data contains duplicate parcel geometries. In such instances, only one entry is retained. Furthermore, if a parcel encompasses multiple raster tiles, only the tile with the lowest cloud coverage was retained for further processing. Thus, in such rare cases, only parts of the parcel’s geometry were included in the polygon clipping and median pixel value calculation described in following step. However, since all subsequent processing and modeling steps relied solely on median pixel values rather than on individual pixels, merging information from multiple tiles is unnecessary.

**Fig. 4:**
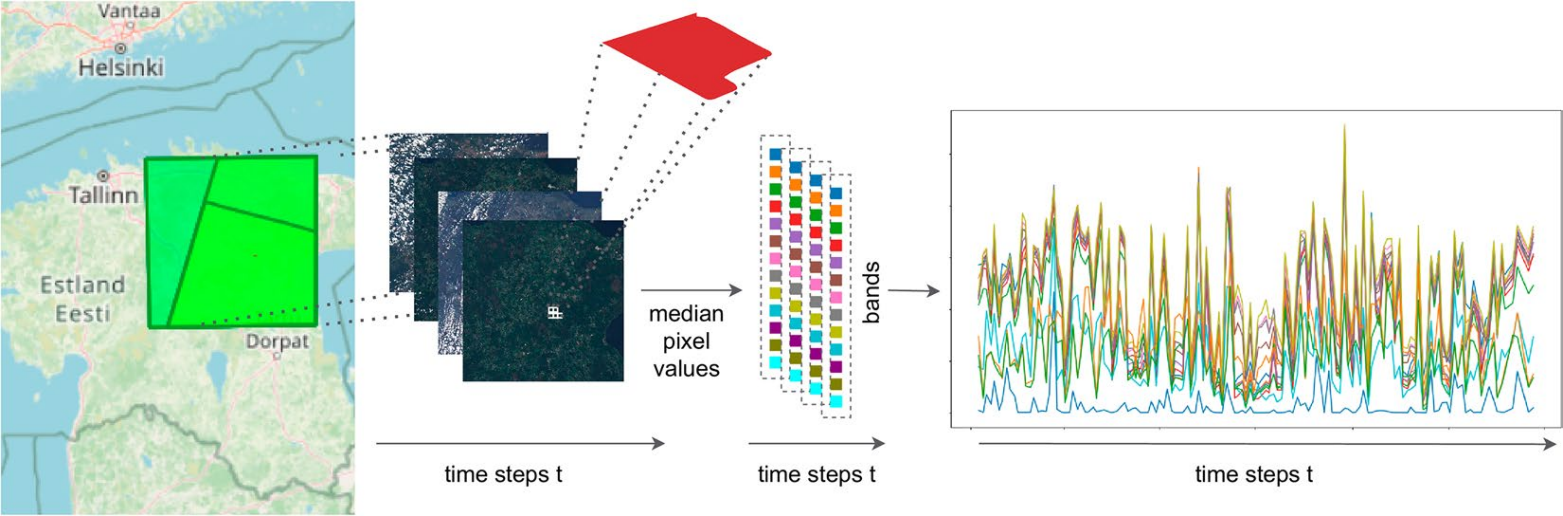
Illustration of the data processing to obtain a median pixel time series from Sentinel-2 raster tiles for a single agricultural parcel. All Sentinel-2 tiles for the year 2021 that overlap with the parcel’s geometry (red polygon) are collected. For each individual time step and Sentinel-2 band, they are clipped to the extent of the polygon before calculating the median pixel value for each time step and band individually, resulting in a multi-spectral time series. The integration of this step into the overall pipeline is shown in Fig. 3.

**Clipping of satellite data and calculation of median pixel values**. Parcels were extracted from the dataset to isolate the satellite images corresponding to each geographical unit. The Sentinel-2 data provides satellite imagery with high spatial resolutions of 10 m, 20 m and 60 m per pixel, depending on the spectral band. The combined Sentinel-2 constellation has a revisit time of five days, resulting in a temporal resolution that allows for a significant amount of observations for each agricultural parcel throughout the year. For every parcel and at each available time step, we calculated the median pixel value for each of the 13 spectral bands in the Sentinel-2 raster tiles. This provides a time series containing optical observations for each parcel. It quantifies the light reflected by the Earth’s surface across various wavelengths over the duration of a year. Our derivation of median pixel time series from Sentinel-2 raster tiles is illustrated in Fig. 4.

**Regional mapping**. For improved geographical accuracy and effective spatial sub-division of the dataset, we employed Eurostat’s *GISCO* database (https://ec.europa.eu/eurostat/web/gisco/geodata/statistical-units/territorial-units-statistics (year 2021, shapefile, polygons (RG), EPSG:4326, scale 01, NUTS level 1-3)) to associate the EuroCrops parcels with their corresponding *Nomenclature of Territorial Units for Statistics (NUTS)* regions. These regions are organized in a hierarchical system from level 1 (large socio-economic regions) to level 3 (detailed local areas). This mapping process generated the *raw* EuroCropsML *dataset* (Item S.1 in the **Data Records** Section), which incorporates parcel geometries, crop type label information, and the associated Sentinel-2 time series.

#### Level II: Data Pre-processing

Additional pre-processing steps were performed to convert the raw EuroCropsML dataset (Item S.1 in the **Data Records** Section) into a ready-to-use ML dataset (Item S.2 in the **Data Records** Section).

**Cloud removal**. As clouds can obscure essential image features, cloud removal plays a crucial role in machine learning in remote sensing, satellite imagery, and geospatial analysis. ML models trained on cloud-covered images risk learning inaccurate patterns or developing biases^16^. Eliminating clouds not only enhances data consistency but can also improve prediction accuracy by preventing spurious correlations that would otherwise degrade the overall model performance. Consequently, models trained on clear images typically generalize better to unseen data^17^. Hence, we performed a cloud removal step following the scene classification approach of the Level-2A algorithm^18^. To identify clouds, we relied on brightness thresholds for the red spectral band (B4). If the median reflectance of the band fell below a prescribed threshold (*t*_1_ = 0.07), it was considered cloud-free and deterministically assigned a cloud probability of 0 %. Conversely, if the value exceeded another prescribed threshold (*t*_2_ = 0.25), it was considered cloudy and we assigned a cloud probability of 100 %. All values between the aforementioned thresholds were linearly interpolated and assigned probabilities between 0 % and 100 %. Consequently, all observations with a cloud probability greater than 50 % (*p* = 0.5) were removed. Data points that only contained time series points with a cloud coverage score greater than 50 %, were removed from the dataset completely.

**File name convention and metadata**. To facilitate data loading during the training of ML models, each data point is stored separately as a NumPy.npz file, accompanied by metadata, such as the observations timestamps and the spatial coordinates of the parcel’s centroid. The naming convention <NUTS3-region>_<parcelID>_<EC_hcat_c>.npz is used for all .npz files, where EC_hcat_c is the EuroCrops HCAT3 crop class code^14,15^ (*cf*. the **Data Records** Section).

### Benchmark Tasks

A main goal of EuroCropsML is to facilitate research focused on knowledge transfer between different geographical regions. We propose two transfer learning scenarios by splitting the dataset into subsets for training (Item S.3 in the **Data Records** Section). These are crucial for evaluating and optimizing the performance of ML models in crop classification. The data splits are:

**Latvia**  → **Estonia** (**LV**  → **EE**) The models are pre-trained on data from Latvia only and then fine-tuned and evaluated on data from Estonia.

**Latvia+Portugal**  → **Estonia** (**LV+PT**  → **EE**) The pre-training is conducted on data from Latvia and Portugal, followed by fine-tuning and evaluation on the same data from Estonia as in the first scenario.

**Fig. 5:**
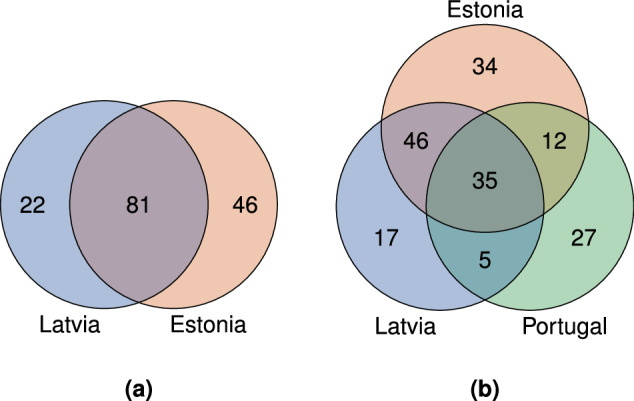
Visualization of annotated crop classes shared between the pre-training and fine-tuning countries.

The two cases are intentionally selected to benchmark algorithms on their performance in geographically and agriculturally distinct and similar regions. Both scenarios contain different classes that are shared or non-shared between the pre-training and fine-tuning regions, as illustrated in Fig. 5. This represents a common challenge in cross-regional knowledge transfer problems. Hence, for both scenarios, we provide two variants of the aforementioned pre-training splits. One that contains all available pre-training classes, while the other only contains classes that overlap with the Estonian data. The latter results in 81 classes for (LV → EE) and 93 classes for (LV+PT → EE).

For the pre-training stage, we downsample the class pasture meadow grassland grass to the median frequency of all other classes. For fine-tuning, we create seven different few-shot learning scenarios simulating challenging real-world use cases of varying data scarcity. In addition to utilizing all available data from the fine-tuning training subset, we provide splits for 1-, 5-, 10-, 20-, 100-, 200-, and 500-shot classification. That is, for a *k*-shot split, for each class in the training set of the fine-tuning data, a maximum of *k* samples is used (or as many as there are available if this is fewer than *k*). The process of generating the splits can be outlined as:

**Pre-train dataset**

1. Filtering pre-processed files for relevant pre-training NUTS-regions
2. Optionally, filtering the pre-processed files to a subset of classes.
3. Downsampling pasture meadow grassland grass class
4. Creating the training-validation split using scikit-learn. 80% are allocated for training, the remaining 20% for validation and hyperparameter tuning.

**Fine-tune dataset**

1. Filtering pre-processed files for relevant fine-tuning NUTS-regions
2. Creating the training-validation-testing split using scikit-learn. 60% are selected for training and 20% for validation and testing, respectively.
3. Sampling 1000 samples from the validation subset
4. Sampling of *k* ∈ {1, 5, 10, 20, 100, 200, 500} samples per class from the training subset

## Data Records

The EuroCropsML dataset (version 11) is publicly hosted on Zenodo^19^. We provide three processing stages of the dataset:

1. **raw data**: The raw Sentinel-2 L1C time series, including all available observations, allowing researchers to perform their own pre-processing steps, *cf*. Section **Level I: Data Acquisition**.
2. **pre-processed data**: The ready-to-use ML dataset after cloud removal, additional data cleaning, and calculation of spatial coordinates of the parcel’s centroid, *cf*. Section **Level II: Data Pre-processing**.
3. **pre-built splits**: Multiple pre-training (training and validation) and fine-tuning (training, validation, and testing) task configurations for benchmarking few-shot crop type classification (*cf*. Section **Benchmark Tasks**) in a transfer learning scenario. This is a decomposed version of Item S.2 and must be used in combination with the pre-processed data.

**Fig. 6:**
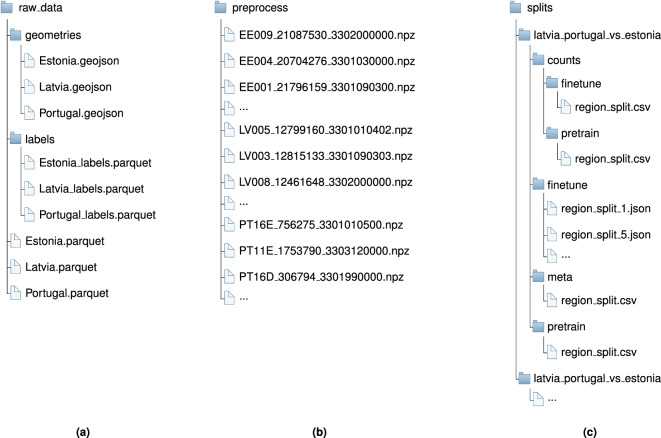
Data records on Zenodo, referring to version 11 of the dataset. (**a**) Raw data (*cf*. Section **Level I: Data Acquisition** and Item S.1 in the **Data Records** Section). The files designated as <Country>.parquet contain the actual cropped time series and reference data. Furthermore, the files named <Country>_labels.parquet contain only the parcel-label matches, thus enabling rapid loading of the labels. (**b**) Pre-processed and ready-to-use dataset for ML (*cf*. Section **Level II: Data Pre-processing** and Item S.2 in the **Data Records** Section). (**c**) Pre-defined data splits used for benchmarking (*cf*. Section **Benchmark Tasks** and Item S.3 in the **Data Records** Section).

Figure 6 shows the structure of the data records on Zenodo, wherein each of the aforementioned stages is represented by a distinct directory. The dataset is organized into individual sub-collections for each country, facilitating the future addition of additional countries.

## Technical Validation

As the ground truth label annotations and parcel geometries are directly sourced from EuroCrops^14^, our validation in the following is limited to the processing of the Sentinel-2 data as well as the benchmarking feasibility of the proposed dataset. As there are no Sentinel-2 ground truths available for the time series, alternative measurements were taken to validate the data processing and serve as sanity checks on several data samples.

### Clipping Process

**Fig. 7:**
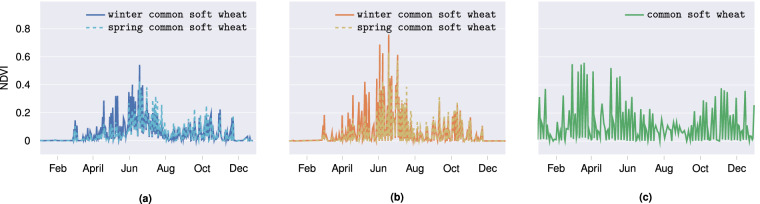
The mean NDVI (taken over all parcels) for winter common soft wheat/spring common soft wheat (Latvia and Estonia) and common soft wheat (Portugal) after cloud removal throughout the year 2021. It should be noted that Portugal does not distinguish between winter and spring wheat. Hence, the mean NDVI may encompass both classes.

Once the clipped median values for each country had been obtained, it was ensured that each parcel had values present. Thereafter, we verified the average NDVI per class and country. The NDVI is a measure of the amount of light reflected by a plant. It is therefore employed in the monitoring of crop growth periods, with higher NDVI values indicating greater crop biomass, and can serve as an instrument for verifying the accuracy of Sentinel-2 time series. Consequently, during the flowering season, we anticipate to observe higher NDVIs, whereas during the planting season, we expect values close to zero. Figure 7, for instance, shows the mean annual NDVI for winter common soft wheat/spring common soft wheat and common soft wheat per country. Winter common soft wheat in Latvia and Estonia is planted before the winter season. The NDVI shows an initial increase during the early stages of spring, reaching its highest values around the onset of summer, which coincides with its flowering season. In contrast, spring common soft wheat is planted in the early stages of spring. Consequently, the NDVI demonstrates a slight delay in its increase in comparison to that observed in winter common soft wheat. In general, winter common soft wheat exhibits slightly higher NDVI peaks than its sibling spring common soft wheat. This is attributable to its head start and the longer growing period it enjoys. However, both crops exhibit analogous biological phases upon entering active growth, resulting in their peaks occurring concurrently.

### Validation of Cloud Removal

**Fig. 8:**
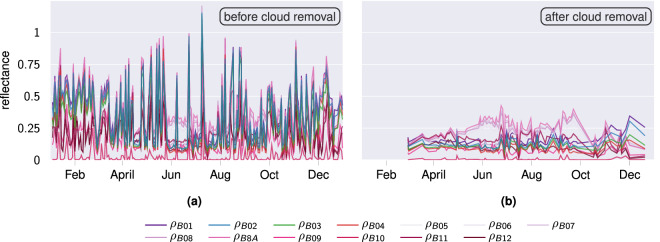
Exemplary Sentinel-2 reflectance data of winter common soft wheat in Estonia. The time series show the three visible light bands (B02 blue, B03 green, B04 red) in their respective color and the remaining bands (B01 aerosol, B05–B8A VNIR, B09–B12 SWIR) in different shades of pink and purple. The high peaks present in the left plot are caused by cloudy observations and are removed in the right plot after applying the cloud removal step.

The cloud removal step is derived from the official scene classification approach of the Level-2A algorithm^18^. Consequently, it has already been validated. However, as only a subset of the complete approach was employed, the method was validated on our data by visualizing the Sentinel-2 time series before and after cloud removal (*cf*. Fig. 8).

### Benchmarking

The integrity of the benchmarking process on our dataset is guaranteed by ensuring that the data intended for the pre-training and fine-tuning stages do not include any overlapping samples. Furthermore, we also ensured that in all cases the pre-defined data split subsets (training/validation/testing) are disjoint. To illustrate the potential use of the EuroCropsML dataset (Zenodo version 11), we provide one example application. This is solely intended as a baseline demonstration and does not represent a comprehensive evaluation study of few-shot learning strategies. Thorough experiments and analyses of various common transfer and few-shot learning algorithms are left to future work.

We pre-train a transformer-encoder architecture with sinusoidal positional encoding as introduced by Vasvani, A. *et al*.^20^ on both use cases outlined in Section **Benchmark Tasks** for a maximum of 150 epochs. The pre-training was terminated if the validation loss did not decrease for more than 15 epochs (early stopping) to prevent overfitting. Following the pre-training phase, the model’s classification head was reset and the model was fine-tuned on the Estonia data in a 1-, 5-, 10-, 20-, 100-, 200-, and 500-shot setting (*cf*. Section **Benchmark Tasks**). Furthermore, we propose a baseline comparison in the form of a randomly initialized transformer model, which has not undergone any pre-training before the aforementioned fine-tuning. The models underwent fine-tuning for a maximum of 200 epochs, with validation occurring after each epoch on 1000 samples of the validation set. Again, the training was stopped in case the validation loss did not decrease for more than five epochs (early stopping). All experiments were conducted using the Adam optimizer^21^ with a tuned learning rate and a batch size of 128.

**Fig. 9:**
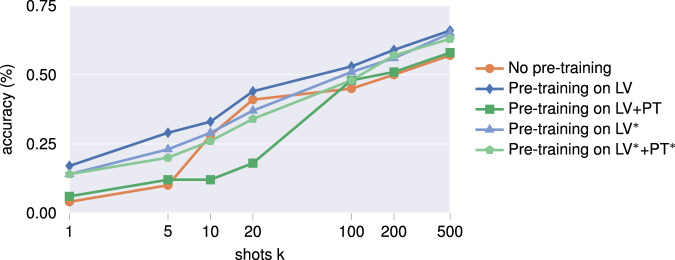
Results of the fine-tuning task, indicating the accuracy (%) obtained on the test set, as also listed in Table 2.

Figure 9 and Table 2 show the classification accuracies on the test set. The model pre-trained on all Latvian data demonstrates superior performance across all settings when compared to the two other approaches. Although the incorporation of data from Portugal generally contributes to the enhancement of the model relative to the baseline, the improvement is not as pronounced as when only pre-training on Latvian data. The restriction of the pre-training classes to those that overlap with the fine-tuning classes, results in a decline in performance when pre-training exclusively on data from Latvia (LV^*^), in comparison to pre-training on the entirety of the Latvian data (LV). Conversely, the removal of non-overlapping classes during pre-training on both Latvia and Portugal (LV^*^+PT^*^) enhances performance in comparison to pre-training on all available pre-training classes (LV+PT). This indicates that pre-training on Latvia in general exploits more characteristics relevant for the subsequent fine-tuning task, demonstrating why regional proximity and similarity can be more valuable than simply increasing data volume. Notably, the analogous climate zones and shared superclasses, as illustrated in Fig. 1, render pre-training on Latvia a valuable approach. Furthermore, for *k* ∈ {10, 20}, the randomly initialized model in fact outperforms pre-training with data from Portugal. The observed accuracies in Table 2 hint at the complexity of our real-world benchmark dataset, making it an ideal resource for evaluating the relative performance of various ML algorithms. The challenging nature of the data enables meaningful differentiation between methodologies and provides insights into algorithm robustness under realistic conditions.

**Table 2:** Test accuracy (%) for the fine-tuning task.

Benchmark task	Shots *k*						
	1	5	10	20	100	200	500
No pre-training	0.04	0.10	0.28	0.41	0.45	0.50	0.57
Pre-training on LV	**0.17**	**0.29**	**0.33**	**0.44**	**0.53**	**0.59**	**0.66**
Pre-training on LV+PT	0.06	0.12	0.12	0.18	0.48	0.51	0.58
Pre-training on LV^*^	0.14	0.23	0.29	0.37	0.51	0.56	0.65
Pre-training on LV^*^+PT^*^	0.14	0.20	0.26	0.34	0.48	0.57	0.63

LV^*^ and PT^*^ denote pre-training limited to those classes that overlap with the Estonia data. Results are also visualized in Fig. 9.

## Data Availability

The code used to generate the dataset is open source and available https://github.com/dida-do/eurocropsml. It is accompanied by a Python package available on the *Python Package Index (PyPI)* and contains pre-defined configuration files which can be used in order to re-process the current dataset.
